# Solubilization of Human Interferon β-1b Inclusion Body Proteins by Organic Solvents

**DOI:** 10.34172/apb.2020.027

**Published:** 2020-02-18

**Authors:** Samira Nekoufar, Ahmad Fazeli, Mohammad Reza Fazeli

**Affiliations:** ^1^Department of Biochemistry, School of Medicine, Iran University of Medical Sciences, Tehran, Iran.; ^2^The Institute of Pharmaceutical Sciences (TIPS), Tehran University of Medical Sciences (TUMS), Tehran, Iran.; ^3^Department of Drug & Food Control, The Institute of Pharmaceutical Sciences (TIPS), Tehran University of Medical Sciences, Tehran, Iran.

**Keywords:** Interferon beta-1b, Inclusion body, Solubilization, Organic solvent, Alcohol

## Abstract

***Purposes:*** Solubilization of inclusion bodies expressed in *E. coli* is a critical step during manufacturing of recombinant proteins expressed as inclusion bodies. So far, various methods have been used for solubilization and purification of inclusion body proteins to obtain active proteins with high purity and yield. The aim of this study was to examine the benefit of organic solvents such as alcohols in solubilization of recombinant interferon β-1b inclusion bodies.

***Methods:*** Effect of important parameters inclusion pH, concentration and type of denaturant and concentration of alcoholic solvents were optimized to formulate a suitable solubilization buffer and investigate their effect on solubilization of interferon β-1b inclusion bodies.

***Results:*** Our findings showed the acidic pH in the range of 2-3 is more suitable than alkaline pH >12 for solubilization and achieving higher content of interferon β-1beta and pure recombinant protein. We have also demonstrated that 1% SDS acts better than 2M urea to solubilize Inclusion body proteins of interferon β-1b at pH of 2-3. The interferon concentration was 2.35 mg per 100 mg IB when we used 40% (*v/v*) 1-propanol and 20% (*v/v*) 2-butanol into the buffer solution as well.

***Conclusion:*** The optimized method provides gentile condition for solubilization of inclusion body at high protein concentration and purity with a degree of retention of native secondary structure which makes this method valuable to be used in production and research area.

## Introduction

Multiple sclerosis (MS) is an inflammatory chronic disease characterized by multiple neurological symptoms and disability which involves the nervous system.^1^ Despite the fact that the exact etiology of MS is far from being completed, the evidence to date has strongly supported that Genetics, environment, and infection (e.g. viruses) may be the factors that are involved in the MS pathogenesis.^1^ So far, interferon B is a drug of choice to alleviate relapsing forms of MS which was discovered for the first time by Issacs and Lindenmann in 1957.^2,3^ This medication is administered either as INFβ-1a; a natural form^1^ or INFβ-1b; a non-glycosylated form.^4-6^ The amount which the human body produces is not sufficient to fully cure the disease, therefore finding another source is necessary. In this regard, the development of biotechnological procedures has paved the way to massive production using bacterial variants.

Of those INFβ-1b is produced in *E. coli* as a host cell. Given that prokaryotic cells are not able to form disulfide bonds and also cannot perform post-translational processes, the expressed recombinant protein does not have its own natural structure as a result and forms intracellular insoluble particles known as “Inclusion Bodies” (IB) which contain native-like secondary structure, condensed, classically inactive and insoluble proteins.^7-9^ In other words, IBs are the result of the recombinant protein overexpression in the expression system.^10,11^ Production of bio-drugs in *E. coli* as a host cell has many challenges due to the formation of IBs. It is necessary to dissolve these intracellular particles using denaturing agents to obtain bioactive proteins.^12^

In spite of new developments in understanding the detailed structure of the IB proteins, there are still many problems with the dissolution and refolding of IB proteins to acquire active proteins.^12^ As indicated, the IBs of the recombinant proteins are typically solubilized at the high concentration of urea and GndHC.^13^ However, this process leads to the destruction of the native-like secondary structure of the recombinant proteins. Therefore, there is a need for the development of alternative methods to solubilize IBs. In this regard, recent studies have revealed that the mild dissolution of IB proteins by anionic detergents such as SDS has resulted in the retention of the secondary structure of the recombinant protein.^9,12^ On the other hand, given the hydrophobic properties of INFβ-1b,^14,15^ this variant has poor solubility in the aqueous solution in the absence of anionic detergents.^15,16^ Thus, it seems that using SDS can be a suitable alternative for urea.

Another evidence suggests that there is an interaction between the organic solvents and proteins.^17^ It has also been shown that the organic solvents such as alkyl alcohols have the ability to promote secondary structure formation at high concentrations.^18,19^ and help to stablize the secondary structure of the proteins.^17^ It has been suggested that the organic solvents provoke transition of proteins to molten globule intermediate states with pronounced secondary and native-like structure. In other words, this is caused by the decrease in dielectric constant of organic solvents.^20^

Because the native-like secondary structure of IB’s proteins, this investigation was designed to study whether organic solvents such as 1-propanol, 2-propanol and 2-butanol as a mild denaturant can be used for solubilization of recombinant proteins of IBs.

## Materials and Methods

### Materials

Inclusion body of recombinant interferon beta-1b was provided by Zistdaru Danesh Company (Tehran, Iran). All chemicals used in this experiment were supplied by Merck (Kenilworth, New Jersey, US) and Sigma Aldrich (St. Louis, Missouri, US).

### Washing

Normally, inclusion bodies contain bacterial contaminations such as DNA and, lipid which need to be minimized, because of their toxic or immunogenic effects on human body. For this purpose, 5 g IBs were washed twice with buffer containing Tris 50mM, EDTA 2mM, TritonX-100 1-2%, NaCl 0.5M, pH = 7 and incubated at room temperature for 30 minutes. The supernatant was discarded by centrifugation at 12,000 rpm for 30 minutes. The resulting precipitate was washed for the third time by a buffer solution containing Tris 50mM, EDTA 2mM, NaCl 0.5M, pH = 7, incubated at room temperature for 30 minutes and centrifuged at 10 000 rpm for 10 minutes. The recovery yield was calculated by weighing the washed pellet divided into the unwashed pellet. The recovery yield resulting from washing step was 2.1 g.

### Solubilization

#### pH optimization

In order to optimize the pH of solubilization, different buffers in two acidic and basic pH were prepared (Table 1). The pH was adjusted to acidic (pH=2) using 0.2% TFA and 37% HCl, as well as alkaline pH (pH=12) which was set by 100 mM arginine. An effective reducing agent was also added constantly to each buffer solution such as dithiothreitol at a concentration of 50mM and a non-polar organic solvent, 2-butanol, at a concentration of 30%. Denaturing agents of urea at the final concentration of 2 M and SDS at the concentration of 1% were used as well (Table 1). An 84 mg Inclusion body were added to each buffer solution and incubated for 3 hours at 37°C. Then they were centrifuged at 10 000 RPM for 10 minutes at 10°C. Each supernatant was analyzed by 14% reduced SDS-PAGE and subsequent densitometric analysis was carried out using Bio Rad Image Labe 6.0 software. A fraction of each supernatant was subjected to Ammonium Acetate precipitation (100mM, pH 5-6) and precipitates were solubilized in 1 milli q water amount of protein in solubilized samples was quantitatively determined by UV absorption.

**Table 1:** Chemical composition for optimizing pH

**Sample Recipe**	**1**	**2**	**3**	**4**	**5**	**6**	**7**	**8**
Arginine (mM)	100	100	100	100	0	0	0	0
TFA (%)	0	0	0	0	0.2	0.2	0.2	0.2
SDS (%)	0	1	0	1	0	1	0	1
Urea (M)	0	0	2	2	0	0	2	2
2-Butanole (%)	30	30	30	30	30	30	30	30

### Optimization of urea and SDS in presence of the organic solvent

According to the Table 2, 12 different buffer solutions were prepared for selecting the optimal denaturant. Briefly, each buffer was set up in either 1% SDS or 2M Urea containing organic solvents at final concentrations of 25% (*v*/*v*) of each of them. As an effective reducing agent, dithiothreitol was added to each buffer solution at a concentration of 50mM. After addition of inclusion body suspension (135 mg) to each buffer solution, the mixtures were incubated for 3 hours at 37°C, and centrifuged at 10 000 rpm for 10 minutes at 10°C. Each supernatant was analyzed by 14% reduced SDS-PAGE and Bio Rad gel documentation image lab Software was applied to analyze of each band. A fraction of supernatant was used for ammonium acetate precipitation. Precipitated proteins were solubilized in 1milli q water and subjected to UV absorption in order to determine protein concentration.

**Table 2:** Optimization of urea and SDS

**Sample Recipe**	**1**	**2**	**3**	**4**	**5**	**6**	**7**	**8**	**9**	**10**	**11**	**12**
SDS (w/v)	1	0	0	0	1	1	0	0	1	1	0	0
Urea (M)	0	2	0	0	0	0	2	2	0	0	2	2
1-Propanole (v/v)	0	0	25	0	25	0	25	0	25	0	25	0
2-Propanole (v/v)	0	0	0	25	0	25	0	25	0	25	0	25
2-Butanole (v/v)	0	0	0	0	0	0	0	0	25	25	25	25

Note: Acidic pH (=2) has been considered in all samples. Alkyl alcohols were added in constantly (25%).

### Optimization of organic solvents

The response surface methodology (version 7.0.0; Stat-Ease, Inc., Minneapolis, Minnesota, US) was applied to optimize a different combinatorial aspect of buffer solution. A User Defined design of experiments, 1-propanol, and 2-butanol, was employed at values of 0-90. The 1% SDS is considered as denaturant agent in each buffer solution (Table 3). Also, each buffer solution was received a dithiothreitol as a reducing agent at the final concentration of 50mM. Following addition of Inclusion body 100 mg into the prepared buffer solutions, they were incubated at 37°C for 3 hours and centrifuged at 10 000 RPM for 10 minutes at 10°C. Supernatants were carefully collected and analyzed by 14% reduced SDS-PAGE and densitometric analysis was carried out by Bio Rad image lab software. A fraction of supernatants was subjected to Ammonium acetate precipitation. Precipitates were solubilized in 1milli q water, absorbance was measured at 280 nm for protein estimation.

**Table 3:** Optimization of organic solvents

**Sample Recipe**	**1**	**2**	**3**	**4**	**5**	**6**	**7**	**8**	**9**	**10**	**11**	**12**	**13**
1-Propanole (v/v)	90	30	60	45	37.5	45	15	37.5	15	15	0	0	0
2-Butanole (v/v)	0	30	15	45	37.5	0	15	15	37.5	60	45	0	90

Note: Acidic pH (=2) has been considered in all samples. The experimental design was carried out by using two factor factorial Design.

### Circular dichroism analysis of purified IFNβ-1bs

The α-helicity of purified IFNβ-1b in different buffer solutions was estimated using the Far-UV spectropolarimeter (Aviv, Lakewood, USA) at the region of 195-260 nm. The precipitated proteins by ammonium acetate were diluted in 50mM phosphate buffer to final concentration of 1 mg/mL, then the CD spectra was measured at 25°C. The spectra were processed using the software supplied with the device. Each sample was screened three times and the average value was plotted.

## Results and Discussion

### The acidic pH for solubility of IFN-β-1b inclusion body proteins

**Figure 1:**
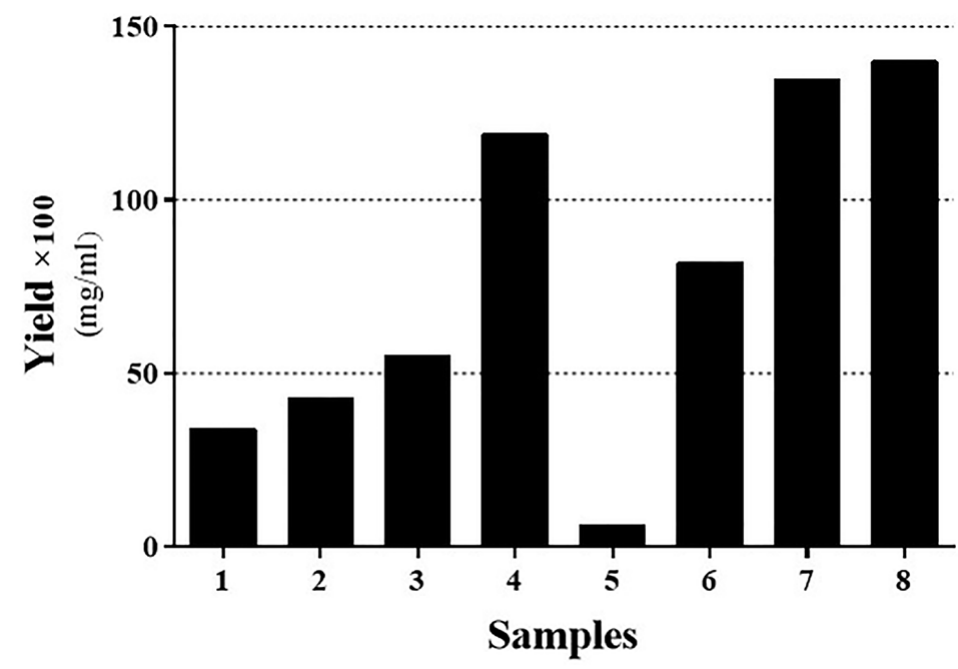
The yield and purity of the IFNβ-1b recombinant protein present in the solubilized Inclusion Body at both acidic and alkaline pH. Samples are based on Table 1. Black and Gray bars represent the yield and purity, respectively. To better insight, the real yield was multiplied by a constant factor (×100).

Eight series of reactions were used to determine the optimum pH condition of either acidic or alkali and urea and SDS during solubilization of inclusion body of interferon beta-1b (Table 1). There is an estimation that organic solvents such as alcohols with less polarity have more effect on solubilizing IB proteins; therefore, 2-butanol was added to the reactions as a constant content.^21^ As Figure 1 indicates, the acidic environment has a higher impact on solubilizing the secondary native-like proteins from inclusion bodies and have yielded more than other buffer solutions (Figure 1; sample no. 6). These results in line with findings of Zhuravko et al who reported that the maximum yield of the proteins was observed at a pH value less than 3. They have also shown that a pH value higher than twelve results in partial degradation of the protein.^21^ In fact, they confirmed the previous results which have shown that the dissolution of IBs at high pH’s can lead to irreversible protein denaturation.^22^ On the other hand, protein dissolution can be both detergent and pH-dependent which indicates the presence of ionic and hydrophilic interactions in IBs.^21^ Moreover, according to findings of Nick Pace et al unfolded protein state requires more net charges to better interact with denaturant agent for dissolution. These net charges are prepared when pH is lowered.^23^ Based on our findings, hereafter, pH = 2 was selected for the downstream optimization process.

### High solubilization of IFN-β-1b IB proteins by SDS

**Figure 2:**
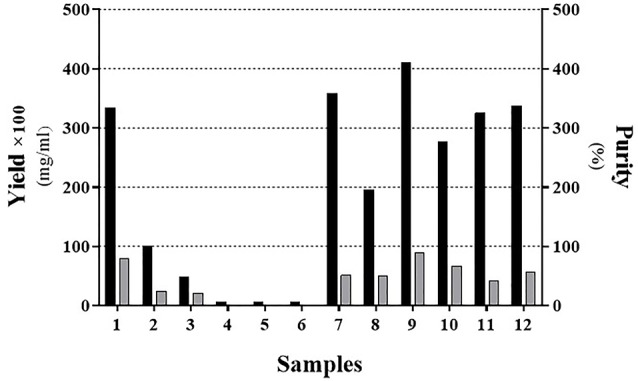
The yield and purity of the IFNβ-1b recombinant protein presents in the solubilized Inclusion Body with/without Urea or SDS. Samples were ordered as indicated in Table 2. Black and Gray bars represent the yield and purity, respectively. To better insight, the real yield was multiplied by a constant factor (×100).

According to Table 2, we showed that mild solubilization is more effective using 1% SDS comparing to the 2M of urea and yielded a purer product (Figure 2; Sample No. 9). Interestingly, buffer solutions containing 2-propanol (Figure 2; Samples No. 4, 6, 8, 10, and 12) has not been a good result compared to other buffer solutions.

Several studies have used GndHCL or urea for the solubilization of recombinant proteins from inclusion bodies.^13^ However, SDS as an anionic detergent was used less frequently.^24^ Traditionally, the chaotropic agents such as urea and GndHCL are used for solubilizing IB proteins.^12^ However, the recent study utilizes such detergents with different concentrations and has demonstrated that increasing the concentration reduces the recombinant protein. They have also revealed that the solubilization of the intended protein by a solution with pH=2.0 and using no urea or GdnHCl has resulted in a maximum release of interferon beta-1b from IB.^21^ Furthermore, according to Vallejo’s findings, dissolution of the inclusion bodies using a detergent such as urea is sensitive to pH. Vallejo also indicates that the optimal pH for protein dissolution should be determined.^22^

### High solubility of IFN-β-1b inclusion bodies in 1-propanol and 2-butanol

As indicated inFigures 3 and 4, the sample No. 8 which contains 1% SDS combined with 1-propanol and 2-butanol has better results and gives high pure content protein. This effect was achieved in the ratio of about 2 :1 of 1-propanol to 2-butanol as shown in Table 3. At first glance, it seems that samples No. 2, 3 and 9 (Table 3) have yielded good results too. However as shown in Figure 4, protein purity and protein content of these samples are less than sample No. 8. We set up another experiment based on Table 4 to have a better characterization of the exact proportion of two organic solvents. Our analysis confirmed that the ratio of 2:1 of 1-propanol to 2-butanol is more effective for dissolution of IFNβ-1b recombinant proteins from insoluble IBs (Figure 5; Sample No. 14) and was yielded high content and pure proteins. Similar to recent work,^21^ our findings indicated that using alcohols with less polarity is effective for the dissolving of insoluble recombinant proteins of IBs. However, in contrary to their results, it seems that effective hydration is provided by the simultaneous presence of two simple alkyl alcohols.

**Figure 3:**
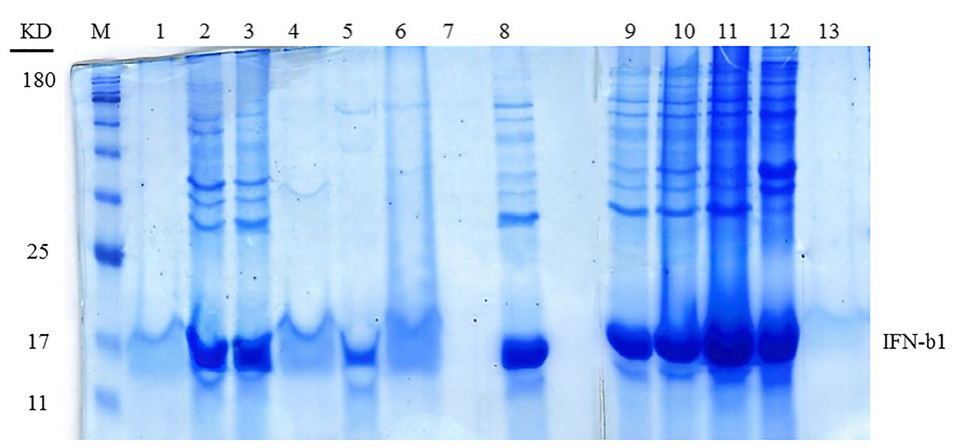
The 14% SDS-PAGE analysis of the solubilized IFNβ-1b Inclusion Body in different concentrations of the organic solvents with 1% SDS. Lane M is the protein ladder in kilodalton (kDa) and subsequent lanes are samples presented in Table 3.

**Figure 4:**
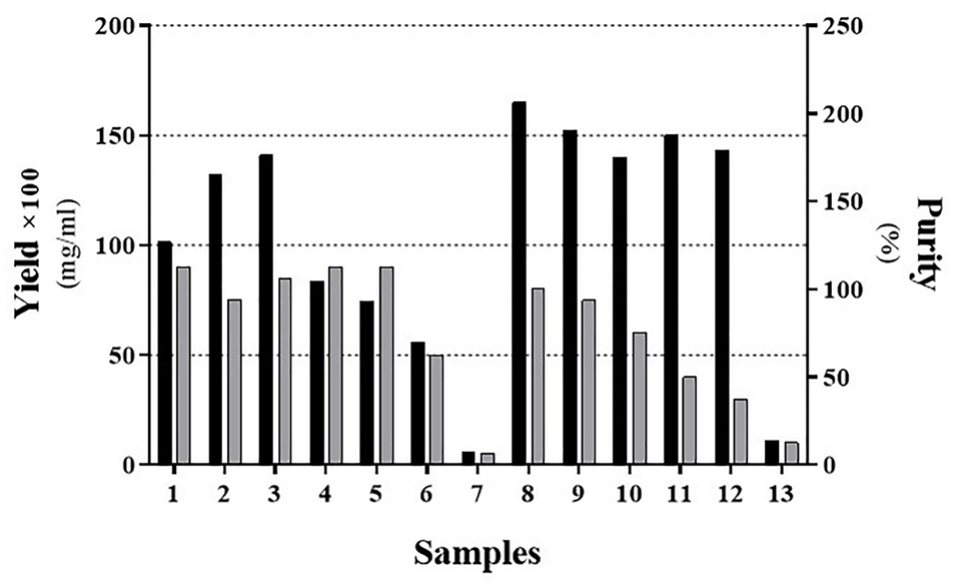
The yield and purity of the IFNβ-1b recombinant protein present in the solubilized Inclusion Body in different concentrations of the alkyl alcohols with 1% SDS. Black and Gray bars represent the yield and purity, respectively. To better insight, the real yield was multiplied by a constant factor (×100).

**Figure 5:**
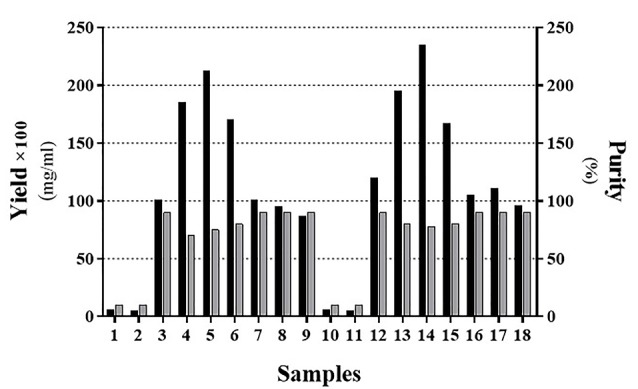
Determination of the exact proportion of 1-propanol to 2-butanol. Samples ordered as indicated in Table 4. Black and Gray bars represent the yield and purity, respectively. To better insight, the real yield was multiplied by a constant factor (×100).

**Table 4:** Determination of the organic solvents’ ratio relative to each other

**Sample Recipe**	**1**	**2**	**3**	**4**	**5**	**6**	**7**	**8**	**9**	**10**	**11**	**12**	**13**	**14**	**15**	**16**	**17**	**18**
SDS (w/v)	1	1	1	1	1	1	1	1	1	1	1	1	1	1	1	1	1	1
1-Propanole (v/v)	20	20	20	20	20	20	20	10	10	0	10	20	30	40	50	60	70	80
2-Butanole (v/v)	0	10	20	30	40	50	60	70	80	20	20	20	20	20	20	20	10	10

Note: Acidic pH (=2) has been considered in all samples.

Given that the hydroxyl group of 2-propanol exhibits less freedom than 1-propanol’s OH-group. Therefore, it showed no proven solubility in all experiments. In fact, the simultaneous effect of the hydroxyl group, the alkyl group and the length of the alkyl alcohol carbons play a major role in the process of protein dissolution. The 1-propanol is easily soluble in water due to having a short allylic chain. On the other hand, the hydroxyl group has more freedom. These properties give its amphipathic effect and resulted in easy solubilization of proteins present in IBs. In the case of 2-butanol, despite the fact that the carbon chain is longer than 1-propanol, the presence of hydroxyl group on its second carbon can properly maintain the hydrophilic and hydrophobic balance in the dissolution process and has an effective impact on the dissolution along with 1-propanol.

### Circular dichroism analysis

**Figure 6:**
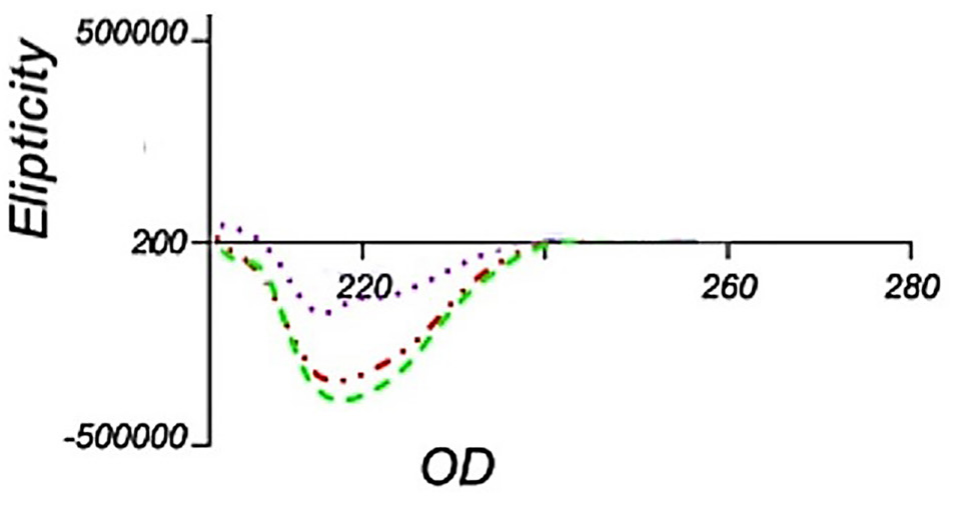
Far-UV CD spectra of IFNβ-1b recombinant proteins obtained in different optimization steps. Green and Purple dotted-dashed lines represent for Samples number 3 and 8 which were selected of Figure 4, respectively as well as the Red dotted-dashed line represents for sample number 14 from Figure 5.

To analysis the secondary structure of solubilized interferon β-1b using CD spectroscopy, we chose samples No. 3 and 8 of Figure 4 as well as sample 14 from Figure 5; because they had the highest purity and protein content. As demonstrated in Figure 6, it seems that the sample No. 3 and 14 have maintained a better secondary structure rather than sample No. 8 and the structure is similar to the structure of the native protein reported elsewhere. However, sample No. 3 has higher α-helix and lower random coil formation in contrast to the sample No. 14 (Supplementary file 1, Table S1). These results suggest that despite the fact that the ratio of 4 to 1 for 1-propanol to 2-butanol has been better in maintaining the secondary structure, the ratio of 2 to 1 has been improved in terms of quantity and purity. Our study shows that increasing the percentage of 1-propanol causes a reduced level of protein content which is similar to recent work.^21^ However, we observed that this loss has been compensating with the maintenance of protein secondary structure.

In the recent years, mild solubilization of IBs has been considered with the preservation of the second structure of proteins. In this regard, Singh et al concluded that using high concentrations of denaturant agents such as Urea leads to the destruction of the second structure of proteins and driving proteins to the form of random coils as well as exposure of the hydrophobic surface.^9^ As a result, compromising of the secondary structure of proteins leads to the interaction of the denaturant proteins and losing bioactivity of proteins.^25,26^

## Conclusion

Mild solubilization of bodies of interferon β-1b using an anionic detergent, SDS, was more effective than urea in acidic pHcompared to alkali pH. Also, inclusion of 40% (*v*/*v)* 1-propanol and 20% (*v*/*v* ) 2-butanol in solubilization buffer containing SDS resulted in highly pure protein at high concentration However, CD analysis demonstrated that better retention of second structure of the interferon β-1b is occurred at 1-propanol concentration of 60% (*v*/*v*) and 2-butanol concentration of 15% (*v*/*v*) in solubilization buffer.

## Ethical Issue

Not applicable.

## Conflict of Interest

There is no conflict of interest to declare.

## Acknowledgments

The authors would like to express their special thanks of gratitude to Zistdaru Danesh Company for kindly providing Inclusion body of IFNβ-1b. As well as, we thank our colleagues, Shayan Abbasi, Farnaz Ghorbanpour, Saeed Heydari, who provided insight and expertise that greatly assisted the research.

## Supplementary File


Supplementary file 1 contains Table S1.

## References

[1] Kieseier BC (2011). The mechanism of action of interferon-beta in relapsing multiple sclerosis. CNS Drugs.

[2] Isaacs A, Lindenmann J (1957). Virus interference I The interferon. Proc R Soc Lond B Biol Sci.

[3] Isaacs A, Lindenmann J (2015). Pillars Article: Virus Interference I The Interferon Proc R Soc Lond B Biol Sci 1957 147: 258-267. J Immunol.

[4] Faulds D, Benfield P (1994). Interferon beta-1b in multiple sclerosis. Clin Immunother.

[5] Buraglio M, Trinchard-Lugan I, Munafo A, Macnamee M (1999). Recombinant human interferon-beta-1a (Rebif®) vs recombinant interferon-beta-1b (Betaseron®) in healthy volunteers. Clin Drug Investig.

[6] (1995). Interferon beta-1b in the treatment of multiple sclerosis:
final outcome of the randomized controlled trial. The IFNB Multiple Sclerosis Study Group and The University of British Columbia MS/MRI Analysis Group. Neurology.

[7] Upadhyay V, Singh A, Jha D, Singh A, Panda AK (2016). Recovery of bioactive protein from bacterial inclusion bodies using trifluoroethanol as solubilization agent. Microb Cell Fact.

[8] Ventura S, Villaverde A (2006). Protein quality in bacterial inclusion bodies. Trends Biotechnol.

[9] Singh SM, Panda AK (2005). Solubilization and refolding of bacterial inclusion body proteins. J Biosci Bioeng.

[10] Sahdev S, Khattar SK, Saini KS (2008). Production of active eukaryotic proteins through bacterial expression systems: a review of the existing biotechnology strategies. Mol Cell Biochem.

[11] Rosano GL, Ceccarelli EA (2014). Recombinant protein expression in Escherichia coli: advances and challenges. Front Microbiol.

[12] Singh A, Upadhyay V, Upadhyay AK, Singh SM, Panda AK (2015). Protein recovery from inclusion bodies of Escherichia coli using mild solubilization process. Microb Cell Fact.

[13] Fischer B, Sumner I, Goodenough P (1993). Isolation, renaturation, and formation of disulfide bonds of eukaryotic proteins expressed in Escherichia coli as inclusion bodies. Biotechnol Bioeng.

[14] Revel M. Interferon-beta: structure, differential actions, and medical applications. In: Leroith D, Bondy C, eds. Growth Factors and Cytokines in Health and Disease. JAI; 1997. p. 433-520. 10.1016/S1874-5687(97)80034-1.

[15] Lin LS, Yamamoto R, Drummond RJ (1986). Purification of recombinant human interferon beta expressed in Escherichia coli. Methods Enzymol.

[16] Rao DV, Ramu CT, Rao JV, Narasu ML, Rao AK (2009). Cloning, high expression and purification of recombinant human intereferon-beta-1b in Escherichia coli. Appl Biochem Biotechnol.

[17] Buck M (1998). Trifluoroethanol and colleagues: cosolvents come of age Recent studies with peptides and proteins. Q Rev Biophys.

[18] Kumaran S, Roy RP (1999). Helix-enhancing propensity of fluoro and alkyl alcohols: influence of pH, temperature and cosolvent concentration on the helical conformation of peptides. J Pept Res.

[19] Munishkina LA, Phelan C, Uversky VN, Fink AL (2003). Conformational behavior and aggregation of alpha-synuclein in organic solvents: modeling the effects of membranes. Biochemistry.

[20] Uversky VN, Narizhneva NV, Kirschstein SO, Winter S, Löber G (1997). Conformational transitions provoked by organic solvents in beta-lactoglobulin: can a molten globule like intermediate be induced by the decrease in dielectric constant?. Fold Des.

[21] Zhuravko AS, Kononova NV, Bobruskin AI (2015). Features of the solubilization of interferon beta-1B from inclusion bodies. Russ J Bioorgan Chem.

[22] Vallejo LF, Rinas U (2004). Strategies for the recovery of active proteins through refolding of bacterial inclusion body proteins. Microb Cell Fact.

[23] Nick Pace C, Grimsley GR, Scholtz JM. Denaturation of Proteins by Urea and Guanidine Hydrochloride. In: Buchner J, Kiefhaber T, ed. Protein Folding Handbook. WILEY‐VCH Verlag GmbH & Co. KGaA; 2005.

[24] Kim PS, Baldwin RL (1982). Specific intermediates in the folding reactions of small proteins and the mechanism of protein folding. Annu Rev Biochem.

[25] Karpusas M, Whitty A, Runkel L, Hochman P (1998). The structure of human interferon-beta: implications for activity. Cell Mol Life Sci.

[26] García-Fruitós E (2010). Inclusion bodies: a new concept. Microb Cell Fact.

