# Supplementary material for: Solubilization of Human Interferon β-1b Inclusion Body Proteins by Organic Solvents
Source: Adv Pharm Bull. 2020 Feb 18;10(2):233–8. doi: 10.34172/apb.2020.027 (PMC7191233; doi:10.34172/apb.2020.027)
Supplement: Supplementary file 1 — contains Table S1. [file apb-10-233-s001.pdf]

Supplementary file 1

Table S1. Secondary structure analysis of selected samples

	**195-260 nm**		
	**Sample 3**	**Sample 8**	**Sample 14**
**Helix**	99.9 %	98.1 %	99.8 %
**Antiparallel**	0.0 %	0.0 %	0.0 %
**Parallel**	0.0 %	0.3 %	0.0 %
**Beta-Turn**	1.3 %	3.2 %	1.4 %
**Rndm. Coil**	0.1 %	2.7 %	0.3 %
**Total Sum**	101.4 %	104.3 %	101.5 %
